# Identification and Expression Profile of *NCED* Genes in *Arachis hypogaea* L. during Drought Stress

**DOI:** 10.3390/ijms25105564

**Published:** 2024-05-20

**Authors:** Ao Chen, Jingyan Li, Heping Wang, Puyan Zhao

**Affiliations:** 1 Guangzhou Dublin International College of Life Sciences and Technology, South China Agricultural University, Guangzhou 510642, China; aochen594666@gmail.com; 2 College of Horticulture, South China Agricultural University, Guangzhou 510642, China; lijingyan0119@163.com (J.L.); 15890700537@163.com (H.W.)

**Keywords:** peanut, 9-cis-epoxycarotenoid dioxygenase, abscisic acid, drought-tolerant, drought-sensitive

## Abstract

Peanut (*Arachis hypogaea* L.) is an important crop that provides essential proteins and oils for human and animal consumption. 9-cis-epoxycarotenoid dioxygenase (NCED) have been found can play a vital role in abscisic acid (ABA) biosynthesis and may be a response to drought stress. Until now, in *Arachis hypogaea*, no information about the *NCED* gene family has been reported and the importance of NCED-related drought tolerance is unclear. In this study, eight *NCED* genes in *Arachis hypogaea*, referred to as *AhNCEDs*, are distributed across eight chromosomes, with duplication events in *AhNCED1* and *AhNCED2*, *AhNCED3* and *AhNCED4*, and *AhNCED6* and *AhNCED7*. Comparative analysis revealed that *NCED* genes are highly conserved among plant species, including *Pisum sativum, Phaseolus vulgaris*, *Glycine max*, *Arabidopsis thaliana*, *Gossypium hirsutum*, and *Oryza sativa*. Further promoter analysis showed *AhNCEDs* have ABA-related and drought-inducible elements. The phenotyping of *Arachis hypogaea* cultivars NH5 and FH18 demonstrated that NH5 is drought-tolerant and FH18 is drought-sensitive. Transcriptome expression analysis revealed the differential regulation of *AhNCEDs* expression in both NH5 and FH18 cultivars under drought stress. Furthermore, compared to the *Arachis hypogaea* cultivar FH18, the NH5 exhibited a significant upregulation of *AhNCED1/2* expression under drought. To sum up, this study provides an insight into the drought-related *AhNCED* genes, screened out the potential candidates to regulate drought tolerance and ABA biosynthesis in *Arachis hypogaea*.

## 1. Introduction

Drought is one of the most serious abiotic stresses, can cause a series of injury symptoms in plants and decline the global crop yields [1,2]. In order to adapt to drought stress, plants have improved their physiological and biochemical metabolism, forming a complex and effective signal network, including ABA biosynthesis pathway [3]. 

In plants, 9-cis-epoxycarotenoid dioxygenase (NCED) function as a pivotal enzyme in abscisic acid (ABA) biosynthesis and can drive the specific cleavage of 9-cis-epoxycarotenoids to produce the essential ABA precursor, xanthoxin [4,5,6]. Long-term studies have shown that the endogenous hormone abscisic acid (ABA) is intricately linked to regulating drought resistance in some plants [7,8,9,10,11,12]. Furthermore, in some plants, *NCED* genes related to ABA synthesis were critical involvement in drought resistance mechanisms [13,14,15,16]. For instance, the overexpression of *OsNCED5* in *Oryza sativa* has been shown to increase ABA levels, enhance tolerance to drought stress, and accelerate leaf senescence [14]. 

The peanut (*Arachis hypogaea* L.), a widely cultivated crop, is an important source of dietary protein and oils [17,18]. The growth and development of *Arachis hypogaeas* are easily affected by drought stress, especially during the seedling stage [19]. In addition, drought is particularly detrimental to *Arachis hypogaea* kernel production [20,21,22,23,24]. Under drought conditions, *Arachis hypogaea* plants exhibit various physiological and morphological alterations such as leaf curling, wilting, and senescence [25]. These changes reflect adaptive strategies to minimise water loss and cope with the hydric stress [25].

Until now, although the diversity, distribution, and regulation of the *NCED* gene family have been investigated in some plant species like *Populus davidiana*, *Gossypium hirsutum*, and *Oryza sativa* and its participation in drought resistance has been explicated [13,14,15], there is limited information on *Arachis hypogaea*. In order to explore the drought-related NCED in *Arachis hypogaea*, this study used bioinformatic methods to identify *Arachis hypogaea NCED* genes and explored their basic characteristics. Additionally, the expression patterns of *AhNCED* genes related to drought stresses between drought-tolerant and drought-sensitive *Arachis hypogaea* varieties were also investigated. Finally, this study emphasised the immediate response of drought-related *AhNCED* genes, screened out the potential candidate to regulate drought tolerance and ABA biosynthesis.

## 2. Results

### 2.1. Identification and Characterisation of AhNCEDs

To identify the *NCED* genes in the *Arachis hypogaea* genome, *NCED* genes from *Pisum sativum*, *Phaseolus vulgaris*, *Glycine max*, *Arabidopsis thaliana*, *Gossypium hirsutum*, and *Oryza sativa* were used as queries to BLAST against the Peanut Genome Resource (PGR) database. A total of eight *AhNCED* genes were identified. The protein sequences of AhNCEDs range from 546 to 642 amino acids, with molecular weights in the range of 6.16~7.14 kD (Table 1). In addition, the theoretical pI of the AhNCEDs varies from 5.66 to 8.49, indicating different charges under different pH conditions (Table 1). Specifically, the average GRAVY value of these proteins was negative, suggesting that they were hydrophilic proteins (Table 1). The instability coefficients of the AhNCEDs range from 31.72 to 43.00, with a maximum of 43.00 for the most unstable NCED protein, indicating that most of them are stable proteins (Table 1). 

**Figure 1:**
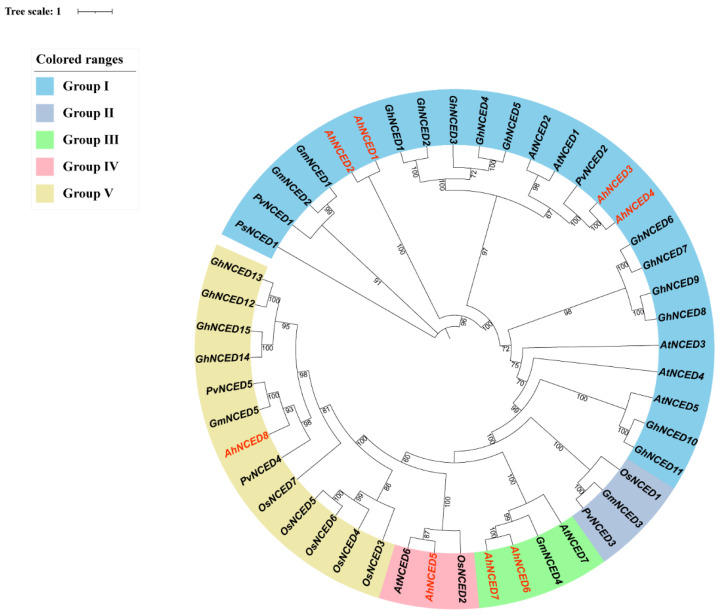
Phylogenetic relationship of the *NCEDs*. Five subgroups (I–V, highlighted with different colours) based on the distribution of genes in the rootless evolutionary tree were divided. *NCED* gene sequences from *Arachis hypogaea* were obtained from the Peanut Genome Resources database (http://peanutgr.fafu.edu.cn/Download.php, accessed on 20 December 2023), while sequences for *Pisum sativum*, *Phaseolus vulgaris*, *Gossypium max*, *Arabidopsis thaliana*, *Gossypium hirsutum*, and *Oryza sativa* were sourced from the NCBI GeneBank. Accession numbers of genes used for alignment and phylogenetic trees are AhNCED1 (AH18G19580.1), AhNCED2 (AH07G23190.1), AhNCED3 (AH03G48330.1), AhNCED4 (AH13G50990.1), AhNCED5 (AH08G22610.1), AhNCED6 (AH10G21080.1), AhNCED7 (AH20G27640.1), AhNCED8 (AH10G21240.1), AtNCED1 (NP_193569.1), AtNCED2 (NP_174302.1), AtNCED3 (NP_188062.1), AtNCED4 (NP_177960.1), AtNCED5 (NP_189064.1), AtNCED6 (NP_191911.1), AtNCED7 (NP_195007.2), GmNCED1 (XP_040874167.1), GmNCED2 (XP_014623805.1), GmNCED3 (XP_003516508.1), GmNCED4 (XP_003522713.2), GmNCED5 (XP_025984636.1), PvNCED1 (XP_007149219.1), PvNCED2 (XP_007144972.1), PvNCED3 (XP_007158058.1), PvNCED4 (XP_007137213.1), PvNCED5 (XP_007137215.1), OsNCED1 (XP_015619611.1), OsNCED2 (XP_015619349.1), OsNCED3 (XP_015611401.1), OsNCED4 (XP_025875749.1), OsNCED5 (XP_025875712.1), OsNCED6 (XP_015648368.1), OsNCED7 (XP_025881983.1), GhNCED1 (XP_016731660.2), GhNCED2 (XP_016699306.1), GhNCED3 (XP_016688155.1), GhNCED4 (XP_016724314.1), GhNCED5 (XP_016683538.1), GhNCED6 (XP_016703654.1), GhNCED7 (XP_016696809.1), GhNCED8 (XP_016714944.2), GhNCED9 (XP_016692107.2), GhNCED10 (XP_016731456.1), GhNCED11 (XP_016670575.2), GhNCED12 (XP_040968868.1), GhNCED13 (XP_016689604.2), GhNCED14 (XP_040971654.1), GhNCED15 (XP_016683086.1), PsNCED1 (XP_050905956.1). Ah: *Arachis hypogaea*; At: *Arabidopsis thaliana*; Gm: *Glycine max*; Ps: *Pisum sativum*; Pv: *Phaseolus vulgaris*; Os: *Oryza sativa*.

In addition, the gene subfamilies and relationships were further investigated. A phylogenetic tree was constructed using NCED protein sequences from *Pisum sativum* (PsNCED), *Phaseolus vulgaris* (PvNCED), *Gossypium max* (GmNCED), *Arabidopsis thaliana* (AtNCED), *Gossypium hirsutum* (GhNCED), and *Oryza sativa* (OsNCED) (Figure 1). The analysis revealed that *NCED* proteins in these species could be classified into five subgroups: I, II, III, IV, and V. Based on the evolutionary analysis of protein sequences (Figure 1), these eight *AhNCEDs* were also classified into four subgroups, labelled as I, III, IV, and V, with four, two, one, and one members, respectively (Table 1). Genes in the same subgroup exhibited high sequence similarity and close genetic relationships, indicating the presence of homologous structures. Notably, the selected proteins displayed a relatively high bootstrap value, particularly in the Fabaceae family, indicating their high conservation among these species (Figure 1). Consequently, the bootstrap values close to 100 in the phylogenetic analysis indicate a high level of confidence in the evolutionary relationship between *AhNCED1* and *AhNCED2* and their close relatives *PsNCED1*, *PvNCED1*, and *GmNCED1–2* within the Fabaceae family (Figure 1). Furthermore, *A. thaliana* in the Brassicaceae family, *G. hirsutum* in the Malvaceae family, and *O. sativa* in the Poaceae family also exhibited a relatively high bootstrap value in these five subgroups, suggesting that the *NCED* gene belongs to an ancient gene family that is conserved across different plant species during their evolutionary differentiation (Figure 1).

### 2.2. Analysis of Conserved Motifs and Domains of AhNCEDs

**Figure 2:**
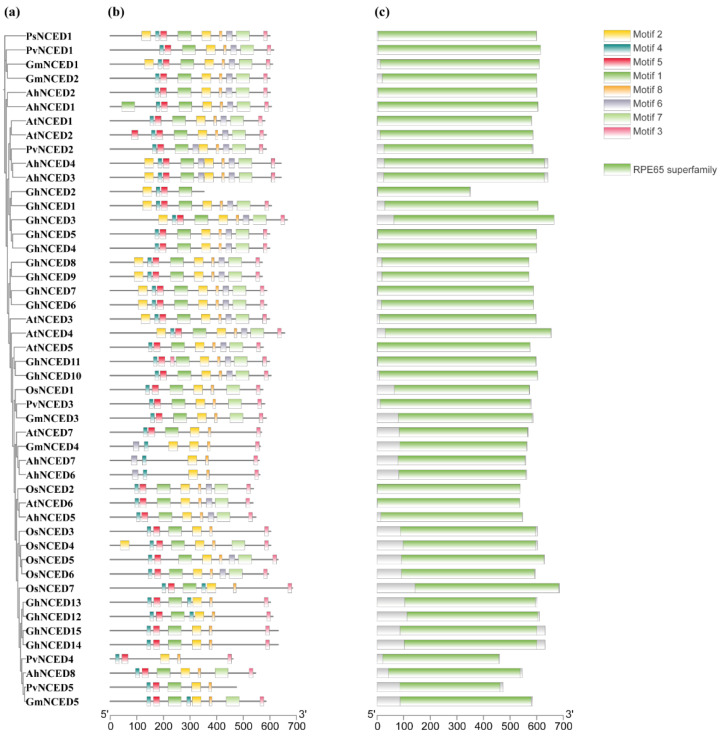
Phylogenetic relationship, conserved motifs, and domains of the NCED proteins. The motifs and domains are arranged schematically based on the pattern of the phylogenetic tree. (**a**) A maximum-likelihood phylogenetic tree was constructed using IQ-tree software v.1.6.8 with the JTT+R5 model and 1000 bootstrap replicates. (**b**) Conserved motifs of NCED proteins were identified using the MEME web server. Different coloured boxes highlight the eight different motifs. (**c**) Domain structure of NCED proteins. Ah: *Arachis hypogaea*; At: *Arabidopsis thaliana*; Gm: *Glycine max*; Ps: *Pisum sativum*; Pv: *Phaseolus vulgaris*; Os: *Oryza sativa*.

Consistent patterns in the quantity and assortment of conserved motifs were observed within each AhNCED protein subgroup. AhNCED1 and AhNCED2 from subgroup I shared eight motifs, AhNCED3 and AhNCED4 from subgroup I shared ten motifs, while AhNCED6 and AhNCED7 from subgroup III each contained five identical motifs (Figure 2). Additionally, the other four AhNCED proteins each displayed a set of seven distinct motifs, specifically motifs 1–5 and motif 8, with AhNCED8 being the exception as it lacks motif 6 (Figure 2). The subgroups’ shared and distinct motif patterns suggest significant structural conservation and specific functions within the AhNCEDs.

**Figure 3:**
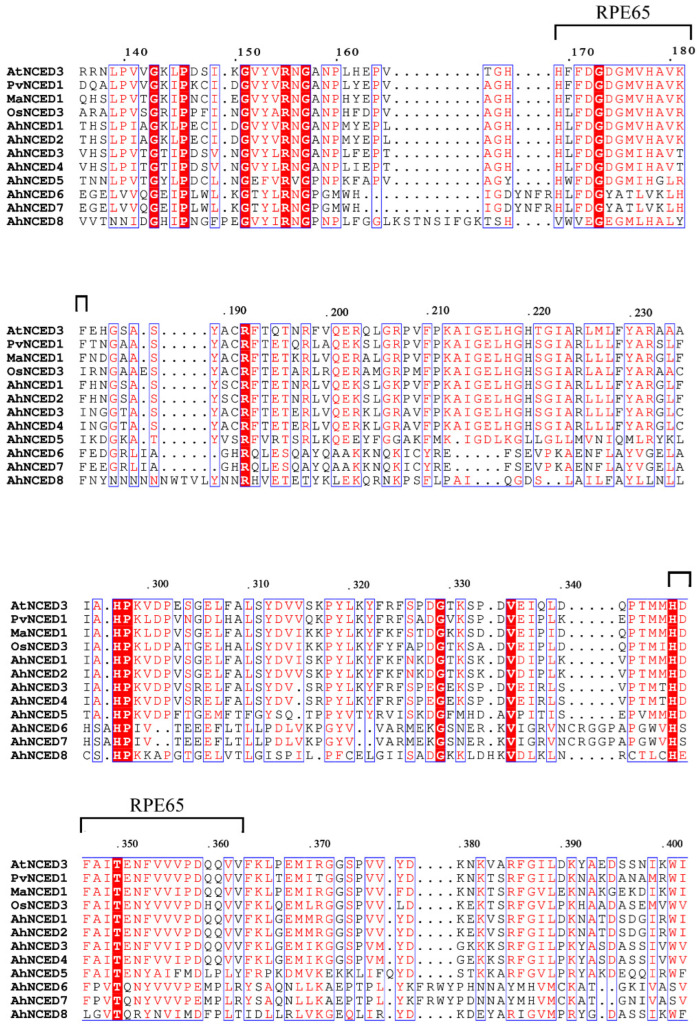
The RPE65 domain in AhNCED proteins. Sequence alignment of eight AhNCED proteins with four species (Arabidopsis thaliana, Morus alba, Oryza sativa, Phaseolus vulgaris) known for their NCED genes [26,27,28,29,30]. The identified RPE65 domains are in black parentheses. Dots represent the positions of the numbers in the protein sequence. Ah: Arachis hypogaea; At: Arabidopsis thaliana; Ma: Morus alba; Os: Oryza sativa.

All AhNCED proteins possess the RPE65 domain, indicating that AhNCEDs are highly conserved across species from different families (Figure 2). The exclusive presence of the RPE65 domain in the NCED family underscores its critical role in the functionality and structural integrity of NCED proteins. The RPE65 domain predominantly occupies positions within the NCED protein sequence (Figure 2), highlighting its importance for the activity and stability of the NCED enzyme. The multiple sequence alignment of AhNCED domains revealed numerous conserved amino acids, particularly histidine (H), glycine (G), and threonine (T), signifying a high degree of conservation in the domain sequences of AhNCEDs (Figure 3).

### 2.3. Gene Structure Prediction of AhNCEDs

**Figure 4:**
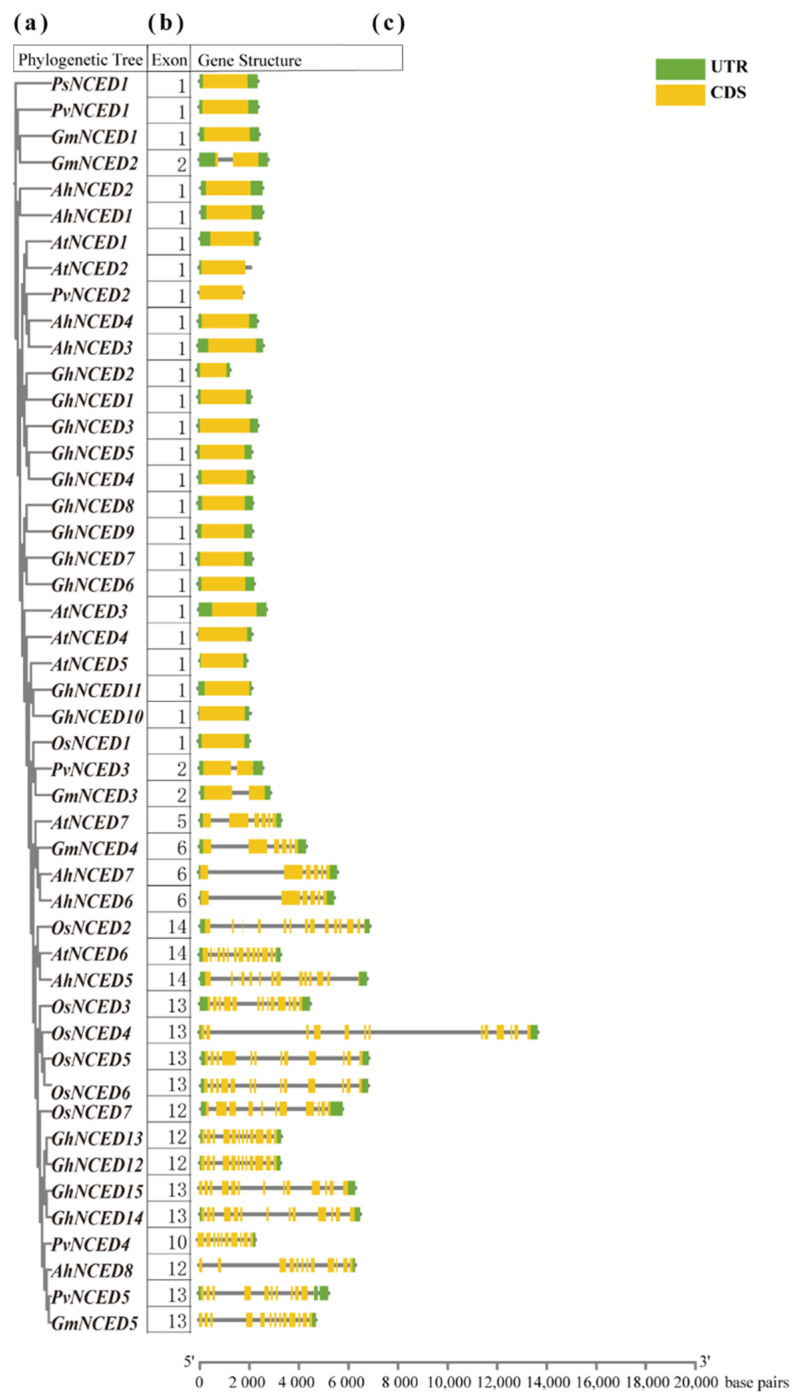
Phylogenetic relationship and gene structure of the *NCED* genes. (**a**) Five subgroups based on the tree shape of rootless evolutionary trees were divided. (**b**) The number of exons in the *NCED* genes. (**c**) The coding sequence and intronic regions are depicted using yellow and green boxes. Ah: *Arachis hypogaea*; At: *Arabidopsis thaliana*; Gm: *Glycine max*; Ps: *Pisum sativum*; Pv: *Phaseolus vulgaris*; Os: *Oryza sativa*.

By comparing with gene structure maps, it is evident that *NCED* genes in different subgroups within the same species exhibit significant differences in the number and length of their exons and introns (Figure 4). Conversely, genes within the same subgroup share similar lengths and have comparable numbers and lengths of introns and exons (Figure 4), resulting in identical gene types. For instance, the subgroups I and II consist of only one or two exons (Figure 4). Interestingly, genes in the same subfamily, such as *AhNCED1*, *AhNCED2*, *PsNCED1*, *PvNCED1*, and *GmNCED1*, possess only a single exon, highlighting a unique structural characteristic within this group (Figure 4). All *NCED* genes in subgroup III contain 14 exons (Figure 4). The *NCED* genes in subgroup III contain five or six exons, while the *NCED* gene in subgroup IV comprises 14 exons (Figure 4). Subgroup V includes thirteen *NCED* genes, with eight genes containing 13 exons, four genes containing 12 exons (*OsNCED7, AhNCED8, GhNCED12, GhNCED13*), and one gene containing 10 exons (*PvNCED4*) (Figure 4). Therefore, the gene structures of the same subgroup exhibit similarities, including the number and length of introns and exons.

### 2.4. Stress-Related Cis-Elements in the Promoters of AhNCEDs

**Figure 5:**
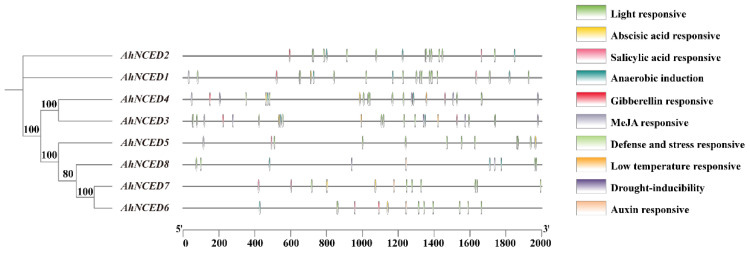
Distribution of hormone responsiveness or stress-related cis-acting regulatory elements in the promoter of *AhNCEDs* (2000 bp upstream region). Ah: *Arachis hypogaea*.

Cis-acting elements located in the promoter regions of genes initiate and regulate gene expression. The analysis revealed that the 2000 bp sequence upstream of the translational start site of each *AhNCEDs* contains 163 cis-acting elements related to hormones and stress responses (Figure 5; Table S2). The potential cis-elements of *AhNCEDs* are implicated in multiple responses to hormones salicylic acid (SA), methyl jasmonate (MeJA), gibberellin (GA), abscisic acid (ABA), auxin (IAA), and stress (light, anaerobic, defence and stress, drought, low-temperature) (Figure 5). Interestingly, nearly all the promoters of *Arachis hypogaeaNCED* genes contained cis-acting elements related to plant hormones and stress responsiveness (Figure 5). Additionally, a large number of SA-related (4.91%), MeJA-related (6.13%), GA-related (3.07%), ABA-related (19.63%), and IAA-related (1.84%) cis-acting elements are found in the *AhNCEDs* promoters (Figure S2). Furthermore, *AhNCED3–4* and *AhNCED8* contained drought-inducible elements, each harbouring four, four and one such element(s), respectively (Figure 5; Table S2). Moreover, a total of 32 ABA-related elements were identified, with *AhNCED1, AhNCED2, AhNCED3, AhNCED4, AhNCED6,* and *AhNCED7* containing the highest numbers of these elements, which were eight, six, five, four, four, and three elements, respectively (Figure 5; Table S2). Further details on the elements and functions of individual genes are listed in Supplementary Table S2.

### 2.5. Chromosomal Localisation of AhNCEDs

**Figure 6:**
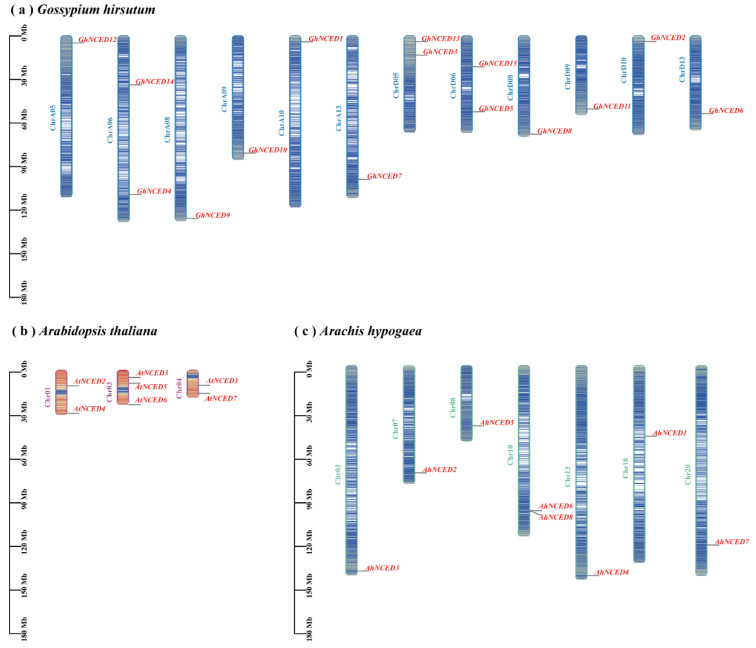
Chromosome localisation of *NCED* genes in *Arachis hypogaea* (green), *Arabidopsis thaliana* (pink), and *Gossypium hirsutum* (blue). Panels (**a**–**c**) depict the gene distribution in each respective species. *Arachis hypogaea* has a total of 40 chromosomes, with *NCED* genes located on 8 distinct chromosomes. *Arabidopsis thaliana* has 10 chromosomes, with *NCED* genes present on 5 of them. *Gossypium hirsutum* has 52 chromosomes, with *NCED* genes on 13 chromosomes. Ah: *Arachis hypogaea*; At: *Arabidopsis thaliana*; Gh: *Gossypium hirsutum*.

For chromosomal location analysis, 38 NCED genes are located on 23 chromosomes, with 12 (*Gossypium hirsutum*), 3 (*Arabidopsis thaliana*), and 8 (*Arachis hypogae*) chromosomes, respectively. The chromosome analysis of the *NCED* gene family across different species revealed an uneven distribution of genes across various chromosomes (Figure 6). For instance, eight *AhNCED* genes were found on seven chromosomes, with Chr10 containing the highest number of *AhNCED* genes (Figure 6). Interestingly, *AhNCED6* and *AhNCED8* were closely clustered on Chr10, indicating possible tandem repeat gene duplication (Figure 6). The *AtNCED* genes exhibited a relatively uniform distribution on chromosomes one, three, and four, whereas in *Gossypium hirsutum*, *NCED* genes are distributed inconsistently across different chromosomes (Figure 6). A total of fifteen *GhNCED* genes were identified on twelve chromosomes (Figure 6). Furthermore, the *NCED* genes in these three species are distributed on three (*Arabidopsis thaliana*), seven (*Arachis hypogae*), and twelve (*Gossypium hirsutum*) chromosomes, with a number that approaches half of the chromosome complement, indicating the widespread distribution of *NCED* genes in the species.

### 2.6. Collinearity Analysis of AhNCEDs

**Figure 7:**
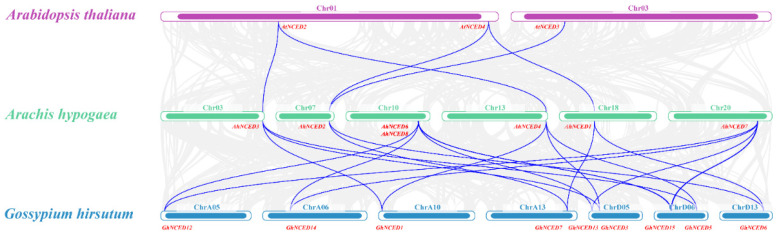
The collinearity relationship among *NCED* genes in *Arachis hypogaea, Arabidopsis thaliana, and Gossypium hirsutum*. The chromosomes of each species are represented by pink (*Arachis hypogaea*), green (*Arabidopsis thaliana*), and blue (*Gossypium hirsutum*) colours. Purple lines indicate the syntenic *NCED* gene pairs between the species, and the highlighted gene names are labelled on the chromosomes. Ah: *Arachis hypogaea*; At: *Arabidopsis thaliana*; Gh: *Gossypium hirsutum*.

Collinearity analysis provides valuable information for identifying homologous gene pairs between species. Using Tbtools-II [31], a multicollinearity analysis of the *NCED* gene family was performed in the model plant *Arabidopsis thaliana*, the Fabaceae family *A. hypogaea*, and the drought-tolerant cotton *Gossypium hirsutum* (Figure 7). Five collinear genes were found between *Arachis hypogaea* and *Arabidopsis thaliana* and eighteen pairs between *Arachis hypogaea* and *Gossypium hirsutum* (Figure 7). The number of *NCED* genes was higher in polyploid species (*Arachis hypogaea* and *Gossypium hirsutum*) than in diploid species (*Arabidopsis thaliana*), indicating that polyploidisation may have contributed to gene family expansion. Most *NCED* genes had one-to-one orthologs between species, but some had multiple orthologs, indicating that they originated from a common ancestor. Collinearity analysis revealed the occurrence of tandem duplications in *Arachis hypogaea* in the collinearity plots (Figure 7), suggesting a potential tandem repeat gene duplication event in *AhNCED1–2* and *AhNCED3–4* as well as *AhNCED6–7*. For instance, *AhNCED2* (on Chr07) and *AhNCED1* (on Chr18) correspond to *AtNCED4*, *GhNCED6*, and *GhNCED7*; *AhNCED3* (on Chr03) corresponds with *AtNCED2*, *GhNCED1*, *GhNCED3*, and *GhNCED5*. In a similar manner, *AhNCED4* (on Chr13) corresponds with *AtNCED2*, *GhNCED1*, *GhNCED3*, and *GhNCED5*; *AhNCED6* (on Chr10) and *AhNCED7* (on Chr20) both correspond with *GhNCED15* (Figure 7 and Figure S3). The sequence alignment confirmed the three pairs of genes initially identified as potential tandem repeats to be three distinct sets of duplicated genes (Figure S3). The collinearity analysis suggests that the *NCED* gene family underwent conservation and diversification during the evolution of *Arachis hypogaea, Arabidopsis thaliana,* and *Gossypium hirsutum*.

### 2.7. Prediction of AhNCED Proteins Structures

**Figure 8:**
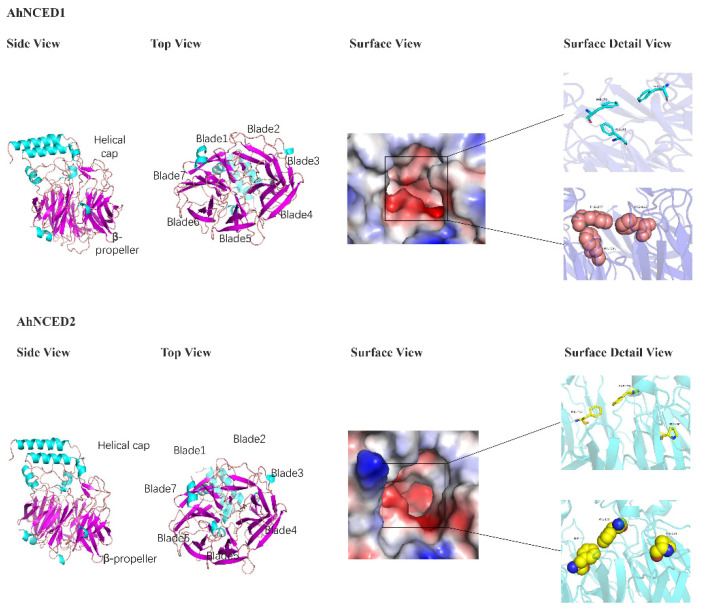
Protein structure simulation diagrams of the 9-cis-epoxycarotenoid dioxygenase 1 and 2 in *Arachis hypogaea* (AhNCED1 and AhNCED2). Blue denotes alpha helices, while purple indicates beta sheets. The red areas represent the active sites of the proteins. Amino acids in the active site pockets are depicted in both stick and sphere formats. These diagrams highlight the structural features of the NCED enzymes in two phenotypically distinct *Arachis hypogaea* cultivars, the drought-tolerant NH5 and the drought-sensitive FH18, which share the same genotype. Ah: *Arachis hypogaea*.

Utilizing VP14 for protein structure simulation, it was observed that the majority of NCED proteins fold into a seven-bladed β-propeller structure, accompanied by four α-helices forming an α-helical domain at the top of the β-propeller (Figure 8). This structure is characterised by conserved features composed of three phenylalanine (Phe) amino acids, with some regions formed by two amino acids (Figure 8 and Figure S4).

### 2.8. Expression Profile of AhNCEDs under Drought Stress

**Figure 9:**
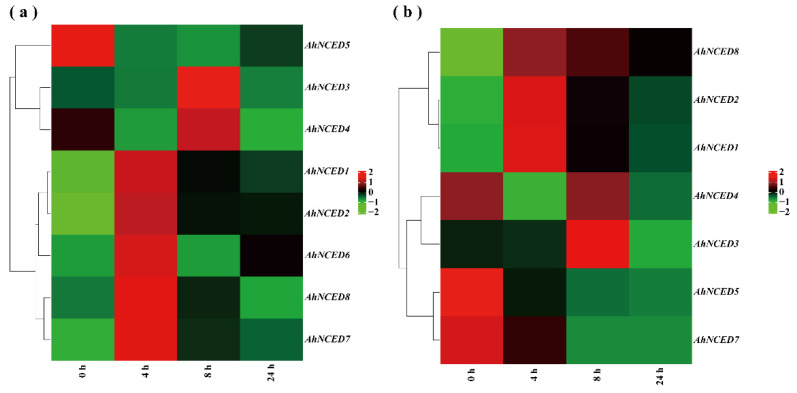
Transcriptome expression analysis of *AhNCED* genes in *Arachis hypogaea* cultivars under drought stress: (**a**) in the drought-tolerant cultivar NH5, and (**b**) in the drought-sensitive cultivar FH18. This heatmap displays the differential expression of *AhNCED* genes over time following drought exposure. The *x*-axis represents the time after drought exposure in hours (h), while the *y*-axis shows the gene names. Expression levels are indicated by a colour scale ranging from −2 (low expression, shown in blue) to +2 (high expression, shown in red). Ah: *Arachis hypogaea*.

The transcriptome data (NCBI database SRA accession number: PRJNA657965) were studied by treating with 20% PEG6000 at time points of 0 h, 4 h, 8 h, and 24 h [32]. It is clear that the expression levels of *AhNCED1* and *AhNCED2* were significantly increased, exhibiting a fold change under 20% PEG6000-induced drought stress (Tables S2 and S3). Specifically, the expression levels of *AhNCED1* and *AhNCED2* gradually decreased as the treatment time extended (Figure 9). The expression levels of *AhNCED6* and *AhNCED8* reached their maximum at 4 h, *AhNCED3* and *AhNCED4* peaked at 8 h, and *AhNCED5* peaked at 0 h. These results suggest that *AhNCED1* and *AhNCED2* may significantly respond to plant drought stress, exhibiting temporal variations in their regulation.

From the results of Heatmaps A and B, *AhNCED1* and *AhNCED2* displayed similar expression patterns under various levels of drought stress, consistently showing higher expression levels. The overall expression levels of *AhNCED3* and *AhNCED4,* and *AhNCED6* and *AhNCED8* were relatively high. In contrast, *AhNCED5* genes exhibited overall low expression levels, with no significant differences between the two *Arachis hypogaea* cultivars (Figure 9). The expression patterns and levels of different genes varied between the two *Arachis hypogaea* cultivars (NH5 and FH18).

### 2.9. Phenotyping of Two Arachis Hypogaea Varieties under Drought Stress

**Figure 10:**
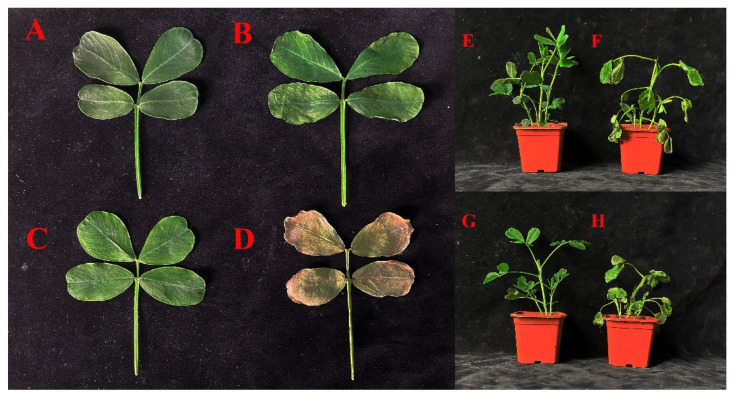
Phenotyping of *Arachis hypogaea* leaves and entire plants under normal and 10-day drought conditions. Drought-tolerant variety (NH5) grown under normal conditions (**A**,**E**), and drought treatment (**B**,**F**). Drought-sensitive variety (FH18) grown under normal conditions (**C**,**G**), and drought treatment (**D**,**H**).

After 10 days of drought treatment, the drought-tolerant *Arachis hypogaea* variety NH5 exhibited minor stress symptoms compared to the control group, which was treated with distilled water. These symptoms included slight leaf curling, indicating its relatively strong drought resistance (Figure 10A,B).

In contrast, the drought-sensitive *Arachis hypogaea* variety FH18 showed significant stress symptoms after the drought treatment compared to the control group. These symptoms were characterised by notable leaf curling and wilting, with leaves becoming dry and brittle and changing colour from green to brown (Figure 10C,D). These changes suggest a poor adaptation of FH18 to drought conditions.

About the overall plants, the 10-day drought treatment led both *Arachis hypogaea* varieties to exhibit phenotypic changes, including a reduction in overall size and thinner stems (Figure 10E–H). However, the stress symptoms of FH18 were more severe than those observed in NH5, further emphasising the significant differences in drought resistance between them.

### 2.10. Expression Analysis of AhNCEDs in Arachis Hypogaea Varieties under Drought Stress

**Figure 11:**
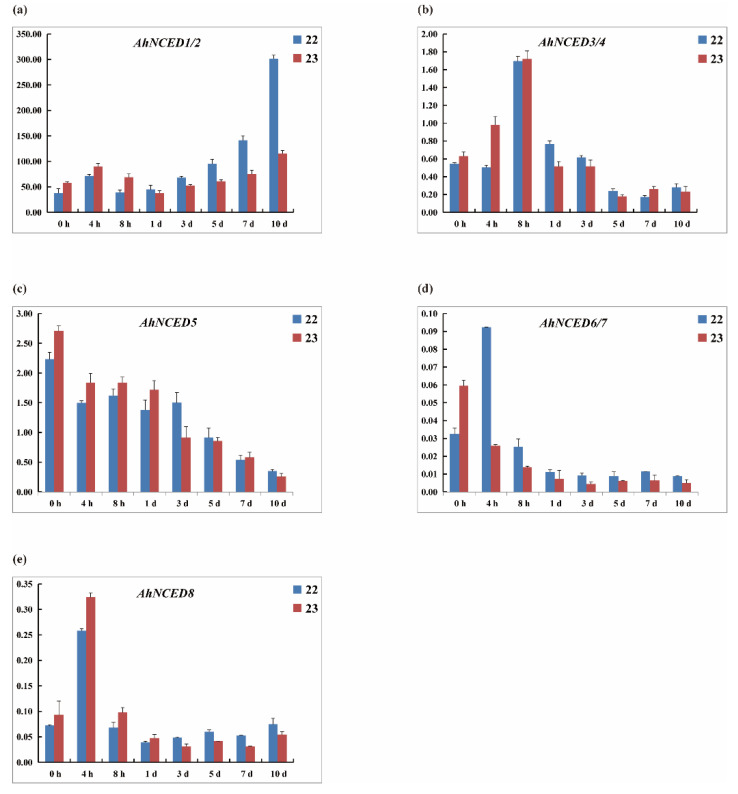
Dynamic expression of *AhNCEDs* in drought-tolerant (NH5) and drought-sensitive (FH18) *Arachis hypogaea* leaves under drought stress. The expression profiles were monitored over 10 days at intervals of 0, 4, 8, 24 h, and 3, 5, 7, and 10 days, highlighting the temporal changes in *AhNCEDs* expression in response to drought conditions. 22: NH5; 23: FH18. Ah: *Arachis hypogaea*.

Owing to the fact that the two genes are in tandem repeats and exhibit a high degree of sequence similarity, it is not feasible to design fluorescent quantitative PCR primers that can distinguish between them. Consequently, the symbol ‘/’ is employed to denote the two individual genes. The quantitative PCR results and heatmap expression profiles were broadly consistent, indicating that *AhNCED1/2* exhibited the most significant expression at all eight-time points, markedly higher than the other six genes (Figure 11b,c). *AhNCED3/4* and *AhNCED5* displayed moderate expression levels, while *AhNCED6/7* and *AhNCED8* showed lower expression levels (Figure 11d,e).

Under drought stress, both NH5 (drought-tolerant) and FH18 (drought-sensitive) varieties showed synchronous expression patterns. The *AhNCED1/2* genes demonstrated notable upregulated expression over the 10 days, whereas *AhNCED3/4* only showed increased expression within the first 8 h, peaked and then declined (Figure 11b). The expression of *AhNCED5* generally decreased over time, while *AhNCED6/7* and *AhNCED8* peaked at 4 h and then fell to an almost non-detectable level (Figure 11c–e).

In both the NH5 (drought-tolerant) and FH18 (drought-sensitive) varieties, the relative expression levels of different genes simultaneously showed consistency across the two varieties. The expression levels in NH5 were initially lower than in FH18 but became higher over time (Figure 11a). For *AhNCED1–4* at 0, 4, and 8 h, the expression levels in NH5 were lower than in FH18, but at the other five time points (1, 3, 5, 7, and 10 days), NH5 exhibited higher expression than FH18 (Figure 11a,b). For *AhNCED5* and *AhNCED8*, in the first four time points (0 h, 4 h, 8 h, 1 d), NH5 had relatively lower expression than FH18 but higher expression in the latter four time points (Figure 11c,e). For *AhNCED6/7*, in the first two time points (0 h, 4 h), NH5 showed relatively higher expression than FH18 but lower expression in the subsequent six time points (Figure 11d).

After the fifth day, different expression levels of the *AhNCED1/2* gene were observed in two *Arachis hypogaea* cultivars. In NH5 (drought-tolerant), the expression levels at 5 days, 7 days, and 10 days were approximately 2.55, 3.77, and 8.04 times higher, respectively, compared to the 0 h timepoint (standard water) (Figure 11a). In contrast, FH18 (drought-sensitive) showed about 1.05, 1.30, and 1.98 times the expression levels at 5 days, 7 days, and 10 days, respectively, compared to 0 h (standard water) (Figure 11a).

## 3. Discussion

A wealth of research has demonstrated the ubiquity of the *NCED* gene family in plants, which are highly conserved across diverse lineages [21]. An unrooted phylogenetic tree was constructed to study the distribution of these genes, which was then classified into five subgroups (I-V) (Figure 1 and Figure S1). The number of motifs, the number of exons in a gene, and the gene lengths were similar within the same subgroup (Figure 2 and Figure 4). Additionally, all *NCED* genes only contain the RPE65 domain. The correspondence between *AhNCED1* and *AhNCED2*, *AhNCED3* and *AhNCED4,* and *AhNCED6* and *AhNCED7* suggests that these genes share a common ancestor and have been retained throughout evolution (Figure 7). These gene pairs belonged to the same subgroup in the phylogenetic tree (Figure 1), and exhibited high similarity regarding their gene structures, conserved motifs, and domains (Figure 2 and Figure 4), indicating that their evolution has been conserved.

Tandem repeat gene duplication occurs in *Arachis hypogaea*. Gene replication refers to a gene duplicating in the genome, which can occur through various mechanisms such as haploid genome polyploidisation, chromosome recombination, and gene transposition [33,34]. This process enhances genome diversity and complexity, providing resources for gene evolution [35,36]. Based on the phylogenetic analysis, It was observed that *AhNCED1* and *AhNCED2, AhNCED3* and *AhNCED4* as well as *AhNCED6* and *AhNCED7*, clustered together (Figure 1), and were found to be homologous to *Arabidopsis thaliana* and *Gossypium hirsutum* in the collinearity analysis (Figure 7 and Figure S3). These results strongly support the occurrence of tandem repeat gene duplication events in *AhNCED1–2* and *AhNCED3–4* as well as *AhNCED6–7*. *NCED* gene replication is not exclusive to *Arachis hypogaea*, as it has also been observed in other plants, such as upland *Gossypium hirsutum* and *Arabidopsis thaliana* [12]. Moreover, duplication events can effectively improve plants’ ability to adapt to various environments [37], and *NCED* gene duplication is likely to enhance drought tolerance in *Arachis hypogaea*. Interestingly, four *NCED* genes (*GhNCED3, GhNCED13, GhNCED5, GhNCED15*) were present on ChrD05 and ChrD06 of *Gossypium hirsutum*, respectively, with duplicated homologs in *Arachis hypogaea* (Figure 7). Therefore, the presence of these genes on the two chromosomes in *Gossypium hirsutum* is likely due to genome duplication events, as *Gossypium hirsutum* and *Arachis hypogaea* may share a common ancestor, and these genes have been retained during evolution. Based on the analysis of conserved structural domains, sequence alignment results, and collinearity analysis, the analysis revealed that *AhNCED* contained only one RPE65 domain and three pairs of tandemly duplicated genes (Figure 2c, Figure 3 and Figure 7; Figure S3). This suggests that a gene family contraction may have occurred in *Arachis hypogaea* during its evolutionary process. Gene duplication events in *AhNCED* genes enhance plant adaptability and offer valuable traits for agricultural biotechnology to improve resilience to climate stress.

The presence of ABA-related and drought-inducibility elements in the promoters of *NCED* genes provides further evidence for their crucial roles in drought response processes. Hormone signalling, particularly ABA, MeJA, and SA, plays a vital role in plant stress resistance [38]. The induction of ABA biosynthesis by *NCED* in response to drought is observed in many other plants, including *Populus davidiana*, *Gossypium hirsutum*, and *Oryza sativa* [13,14,15]. In *A. hypogaea*, *AhNCED1* and *AhNCED2* possess the highest number of ABA-related elements, witsix eight and 6 elements, respectively (Figure 5; Table S2). In addition, *AhNCED1* contains two MeJA elements and one SA element, while *AhNCED2* contains one SA element (Table S2). ABA can interact with hormones such as MeJA and SA [16]. Therefore, the expression levels of *AhNCED1* and *AhNCED2* were significantly upregulated under 20% PEG6000-induced drought stress in the NH5 (drought-tolerant) and FH18 (drought-sensitive) cultivars (Figure 9). These genes exhibit high expression levels in the heatmaps, confirming their essential roles in defence responses to various stresses and plant hormones (Figure 9). Understanding the interaction between *AhNCED* genes and ABA synthesis can aid in developing crops with superior drought tolerance, addressing the increasing global frequency of drought conditions.

The *AhNCEDs* responds to drought stress, exhibiting differential expression patterns among its members and across cultivars. Specifically, we observed temporal differences in the expression levels of the *NCED* genes. Notably, *AhNCED1–*2 showed a gradual decrease in expression (Figure 9), yet its expression exhibited a high fold change in drought-tolerant NH5 and drought-sensitive cultivar FH18 (Tables S3 and S4). Interestingly, the fold changes in NH5 were less pronounced than those in FH18, suggesting that NH5 may not require as high expression levels of these genes to cope with drought stress. This pattern mirrors observations in other plant species, where drought-tolerant cultivars often exhibit lower expression levels than drought-sensitive ones. For example, the *StDRO1* gene in potato and the asparagine synthase and *AMPD* genes in rice display similar trends [39,40]. Due to the identical amino acid sequences in the promoter regions, approximately 300 bp upstream, of *AhNCED1*/*2*, *AhNCED3*/*4*, and *AhNCED6*/*7*, three pairs of similar primers were designed (Table S4). Based on qRT-PCR results, a significant finding of our study is the temporal dynamics of gene expression in NH5 compared to FH18. Initially, *AhNCED* genes in NH5 showed lower expression levels than in FH18. However, as the stress period progressed, NH5 demonstrated a relative increase in expression levels, particularly for *AhNCED1*/*2* (Figure 11). This pattern, where NH5 starts with lower expression but eventually surpasses FH18, indicates a delayed but robust activation of drought response mechanisms in the drought-tolerant variety (Figure 11).

This study underscores the crucial role of *AhNCED1* and *AhNCED2* in Huayu2′’s drought tolerance, with significant upregulation observed under drought conditions (Figure 9b and Figure 11). These two genes exhibited the highest expression increase among the *AhNCEDs*, particularly in NH5, highlighting their crucial role in enhancing drought resistance (Figure 9b and Figure 11). In contrast, the drought-sensitive FH18 showed more moderate gene expression changes (Figure 9b and Figure 11). Post 10-day drought treatment, phenotypic analysis revealed minor stress symptoms in NH5, like slight leaf curling, aligning with its strong genetic drought resistance (Figure 10A,B). In contrast, FH18, a drought-sensitive variety, exhibited severe stress symptoms, including leaf curling, wilting, and discolouration (Figure 10C,D), correlating with lower *AhNCED*1/2 expression. Both varieties showed phenotypic changes like reduced size and thinner stems (Figure 10E–H), but FH18 symptoms were more severe, highlighting the distinct drought resistance between the varieties. These results emphasise that *AhNCED1* and *AhNCED2* are important in drought tolerance and shed light on the genetic basis of drought resistance in *Arachis hypogaea*, supporting efforts to genetically improve crops for greater resilience against climate change.

## 4. Materials and Methods

### 4.1. Plant Material and Treatments

The seeds of *Arachis hypogaea* cultivars (NH5 and FH18, also named Huayu22 andHuayu23, respectively) was gifted by Ms. Lina Yang from the College of Geography and Oceanography at Minjiang University, Fuzhou, Fujian Province, China. *Arachis hypogaea* seeds were sowed and cultivated in the greenhouse at a 23 ± 2 °C, under long-day conditions featuring a 16 h light/8 h dark cycle, with a light intensity of 300 μmol m^−2^ s^−1^. After 21 days, *Arachis hypogaea* seedlings were chosen for water interruption treatment and foliar application of 20% PEG6000. Samples were collected at different time points (0 h, 4 h, 8 h, 24 h, 3 d, 5 d, 7 d, 10 d) [32]. Then, the samples were either used immediately or shock-frozen in liquid nitrogen and stored at −80 °C.

### 4.2. Sequence Retrieval

The protein sequence of NCEDs from *Pisum sativum, Phaseolus vulgaris, Glycine max, Arabidopsis thaliana, Gossypium hirsutum*, and *Oryza sativa* were used as reference sequence targeted to blast the candidate NCEDs in the Peanut Genome Resource (PGR) database (http://peanutgr.fafu.edu.cn/Download.php, accessed on 18 December 2023) and NCBI database (https://blast.ncbi.nlm.nih.gov/, accessed on 18 December 2023). Additionally, the ExPASy web tool was employed to predict parameters such as length, molecular weight (MW), isoelectric point (pI), and grand average of hydropathicity (GRAVY) for the protein sequences (https://www.expasy.org/, accessed on 20 December 2023).

### 4.3. Phylogenetic Analysis of NCED Protein Sequences

The domain architecture of NCED protein sequences was analysed using the National Center for Biotechnology Information Conserved Domain Database (https://www.ncbi.nlm.nih.gov/cdd, accessed on 20 December 2023). All amino acid sequences lacking the required conserved structure were removed from the original sequence, resulting in a comprehensive list of *NCED* gene family members. Based on protein domain prediction results, a total of 48 protein sequences, including 8 AhNCED, 1 PsNCED, 5 PvNCED, 5 GmNCED, 7 AtNCED, 15 GhNCED and 7 OsNCED protein sequences, were retrieved from the NCBI database. Multiple sequence alignment of these 48 NCED protein sequences was performed using IQ-tree (http://www.iqtree.org/, accessed on 21 December 2023), and a maximum likelihood (ML) phylogenetic tree was constructed using the IQ-tree with JTT+R5 model (best-fit model) and 1000 bootstrap test. A *Pisum sativum* NCED protein sequence was selected as an outgroup to determine the evolutionary relationship between the *NCED* genes. The *NCED* genes were named based on their position on the phylogenetic tree. The resulting phylogenetic tree was visualised using the Interactive Tree of Life (iTOL) web tool [41].

### 4.4. Protein Motif and Domain Analysis and Multiple Sequence Alignments of AhNCEDs

The conserved domains of NCED proteins were detected using CDD while identifying *NCED* genes. Full-length protein sequences were analysed using the MEME web to identify conserved motifs. All the gene structures of *NCED* genes were identified and visualised using TBtools-Ⅱ [31] based on the genome annotation gff3 files. To identify conserved motifs in *NCED* genes, the MEME v5.5.1 web server was employed using Zoop for site distribution and setting the maximum number of motifs to 8. Finally, the phylogenetic tree, conserved motifs, and conserved domains were combined and visualised using the Gene Location Visualise Advanced of TBtools-II (South China Agricultural University, Guangzhou, China) [31]. The software also envisioned and merged genetic results and phylogenetic trees. Multiple sequence alignments were constructed using MEGA software using the ClustalW algorithm [42] and manually verified using the ESPript 3.0 web tool (http://espript.ibcp.fr/ESPript/ESPript/, accessed on 20 December 2023). Identifying the RPE65 domain and annotating the multiple sequences were conducted as previously described [29].

### 4.5. Evolutionary Analysis of NCED Genes

The genome annotation GFF3 files of *Pisum sativum*, *Phaseolus vulgaris*, *Glycine max*, *Arabidopsis thaliana*, *Gossypium hirsutum*, and *Oryza sativa* were downloaded from the NCBI database. And the genome annotation GFF3 files of A. hypogaea were downloaded from the Peanut Genome Resource (PGR) database (http://peanutgr.fafu.edu.cn/Download.php, accessed on 20 December 2023). TBtools-II [31] was used to analyse the structure of *NCED* genes in each species in combination with the evolutionary tree. The NCBI GenBank database (https://www.ncbi.nlm.nih.gov/genbank/, accessed on 20 December 2023) was also used to retrieve the exon quantity of the *NCED* genes. Adobe Illustrator software v26.0.3 (64-bit) was used for visualisation.

### 4.6. Cis-Elements Analysis of AhNCEDs Promoters

To identify stress-related cis-acting regulatory elements in the promoter sequences, the 2000 bp upstream regions (from the translation starting sites) of the *NCED* genes were extracted and examined using the PlantCARE web tool (http://bioinformatics.psb.ugent.be/webtools/plantcare/html/, accessed on 21 December 2023). Predictions were made for ten stress-related cis-elements associated with the hormones salicylic acid (SA), methyl jasmonate (MeJA), gibberellin (GA), abscisic acid (ABA), auxin (IAA), and stress (light, anaerobic, defence and stress, drought, low-temperature). The *PsNCED1* gene was also included as an outgroup to expand the analysis. Multiple sequence alignment of the eight *Arachis hypogaea* NCED protein sequences was performed using IQ-tree (http://www.iqtree.org/, accessed on 21 December 2023), and a maximum likelihood (ML) phylogenetic tree with statistical support was constructed using the VT+I model (best-fit model) and 1000 bootstrap test. The cis-acting elements and the phylogenetic tree were then used to generate a figure using TBtools-II [31], which visualises the relationship between the *NCED* gene family and the stress-related cis-acting regulatory elements in the promoter sequences.

### 4.7. Chromosomal Localisation of AhNCEDs

The Gene Density Profile function of TBtools-II was utilised with a bin size of 100,000 and the genome annotation GFF3 files to obtain the gene density information of *Arachis hypogaea*, *Arabidopsis thaliana*, and *Gossypium hirsutum*. The gene location visualisation function of TBtools-II [31] was used to determine the chromosomal locations of *NCED* genes in these three species, with the genome annotation GFF3 files. The results were then plotted on the chromosome location of *NCED* genes, and the chromosome blanks were filled with gene density information.

### 4.8. Synteny Analysis of AhNCEDs

The genome annotation files of *Arachis hypogaea*, *Arabidopsis thaliana*, and *Gossypium hirsutum* were obtained from the NCBI database (https://www.ncbi.nlm.nih.gov/assembly/, accessed on 20 December 2023). To explore the synteny relationship of *NCED* genes among different species, synteny analysis was performed using the One Step MCScanX module of TBtools-II [31], with an E-value threshold of 10^−10^ and five retained blast hits. Collinear gene pairs were identified using the BLASTP algorithm with an E-value threshold of 10^−10^, and the results were visualised using the Multiple Synteny Plot module of TBtools-II [31]. The Clustal Omega website (https://www.ebi.ac.uk/Tools/msa/clustalo/, accessed on 22 December 2023) was used to perform multiple sequence alignment of nucleotide sequences of the tandem repeat sequences.

### 4.9. Modelling of AhNCEDs Proteins

Using PDB: VP14 (GeneBank database number: AAB62181.2) as a template and employing SWISS-MODEL (https://swissmodel.expasy.org/interactive, accessed on 23 December 2023), a homology model of NCED was constructed, and tertiary structure predictions were conducted for the NCED protein sequences identified in the transcriptome [43]. Based on the target–template alignment, the highest-quality templates were determined and selected for model construction [43]. The protein structures, along with the pocket amino acids, were visualised using PyMOL (https://pymol.org/2/, accessed on 23 December 2023).

### 4.10. Heatmap of AhNCEDs Gene Expression Patterns

Furthermore, two A. hypogaea varieties, NH5 and FH18 were sourced from 23 main commercial *Arachis hypogaea* cultivars for physiological characterisation and transcriptomic analysis [32]. The SRA data for NH5 and FH18 under drought stress were obtained from the NCBI database (SRA accession number: PRJNA657965), and the FPKM (fragments per kilobase million) values were extracted for *AhNCED* gene expression analysis [32]. The heat map values of *AhNCEDs* were visualised using the online website Omicshare (http://www.omicshare.com, accessed on 23 December 2023).

### 4.11. RNA Extraction and Gene Expression Analysis

The second leaves of NH5 and FH18 were subjected to drought stress using 20% PEG6000. The experiment spanned eight time points: 0, 4, 8, 24 h, and 3, 5, 7, and 10 days. Each time point was replicated three times to ensure statistical reliability. The protocol extracted RNA from the *Arachis hypogaea* tissues using the Papure Plant RNA kit (Magen Biotech, Guangzhou, China). RNA integrity was confirmed by agarose gel electrophoresis, and quantification was performed using a Thermo NanoDrop 2000 spectrophotometer (Thermo Fisher Scientific, Waltham, MA, USA). Subsequently, RNA was reverse-transcribed into cDNA using HiScript II Q RT SuperMix for qPCR (+gDNA wiper) (Vazyme, Nanjing, China). The qPCR primers were listed in Table S4 and were designed using the Primer3Plus website (https://www.primer3plus.com/index.html, accessed on 23 December 2023). qPCR was conducted on a Bio-Rad CFX96 Real-Time PCR System (BioRad, Hercules, CA, USA), following this program: an initial denaturation at 95 °C for 3 min, followed by 40 cycles of 95 °C for 10 s and 60 °C for 30 s. Actin was the internal reference gene; each sample was analysed in triplicate. Data were processed using the 2^−ΔΔCt^ method [44].

Furthermore, parts of the NH5 and FH18 plants were subjected to a 10-day whole-plant drought stress treatment using 20% PEG6000 (Sangon Biotech, Shanghai, China). After this period, leaves were collected to observe phenotypic changes. Additionally, morphological changes in NH5 and FH18 were assessed and compared between the control group (standard water) and the drought stress group (subjected to a 10-day whole-plant drought treatment using 20% PEG6000).

### 4.12. Statistical Analysis

Statistical analyses were conducted utilizing SPSS software (version 26.0, SPSS Institute, USA). Comparative evaluations of mean values derived from the various treatments were executed via Student’s t-test. Significant disparities are denoted by asterisks, with *, **, and *** signifying *p* values less than 0.05, 0.01, and 0.001, respectively, indicating increasing levels of statistical significance.

## 5. Conclusions

This research identified eight *NCED* genes in *Arachis hypogaea*, analysed their phylogenetic relationships, conserved protein motifs and domains, gene structures, cis-acting elements, and chromosomal localisation and carried out synteny analysis. The presence of ABA-related and drought-inducible elements in the promoters of *AhNCEDs* underscores their crucial roles in drought response. Notably, tandem repeat gene duplication in *Arachis hypogaea*, particularly in *AhNCED1* and *AhNCED2*, *AhNCED3* and *AhNCED4*, and *AhNCED6* and *AhNCED7*, suggests an evolutionary adaptation. Moreover, the research revealed that *AhNCED1* and *AhNCED2*, markedly upregulated in both NH5 (drought-tolerant) and FH18 (drought-sensitive) cultivars, play a vital role in the rapid drought response of *Arachis hypogaea*. Additionally, compared to the FH18, the NH5 variety exhibits a delayed yet robust activation of drought response mechanisms, which was particularly evident in the *AhNCED1* and *AhNCED2*. In summary, this research provides valuable insights into the *NCED* genes of *Arachis hypogaea*, contributing to future efforts to enhance the agronomic traits of *Arachis hypogaea* plants.

## Figures and Tables

**Table 1:** Characteristics of the eight AhNCEDs in *Arachis hypogaea* L. The protein sequences of NCEDs from *Arachis hypogaea* were used as reference sequences to target and blast the candidate NCEDs in the Peanut Genome Resource (PGR) database (http://peanutgr.fafu.edu.cn/Download.php, accessed on 20 December 2023). Ah: *Arachis hypogaea*.

Protein	Accession Numbers	Encoding Amino Acid No.	Molecular Weight (kD)	Theoretical (pI)	GRAVY	Instability Index	Aliphatic Index
AhNCED1	AH18G19580.1	605	6.71	8.49	−0.38	42.04	75.44
AhNCED2	AH07G23190.1	601	6.68	8.49	−0.39	42.98	75.12
AhNCED3	AH03G48330.1	642	7.12	6.39	−0.40	39.93	76.68
AhNCED4	AH13G50990.1	642	7.14	6.39	−0.41	41.83	77.13
AhNCED5	AH08G22610.1	547	6.16	5.66	−0.32	31.72	82.10
AhNCED6	AH10G21080.1	562	6.27	6.99	−0.34	42.71	79.27
AhNCED7	AH20G27640.1	559	6.22	7.59	−0.36	43.00	77.26
AhNCED8	AH10G21240.1	546	6.17	5.54	−0.26	35.49	82.25

## Data Availability

All data are presented in this article.

## Supplementary Materials

The following supporting information can be downloaded at: https://www.mdpi.com/article/10.3390/ijms25105564/s1, Figure S1. Phylogenetic relationship of NCEDs. Figure S2. Different bar lengths in the chart represent the number of various functional cis-acting elements. Figure S3. Three pairs of tandem duplicated *AhNCEDs*. Figure S4. Protein structure modelling of the AhNCED3–8 proteins. Table S1. Cis-acting elements of *AhNCEDs*. Table S2. Fold change of *AhNCEDs* expression in drought-tolerant variety (NH5) under drought stress treatment. Table S3. Fold change of *AhNCEDs* expression in drought-sensitive variety (FH18) under different time points of drought stress treatment. Table S4. Primers for quantitative PCR.
