# Supplementary material for: Identification and Expression Profile of NCED Genes in Arachis hypogaea L. during Drought Stress
Source: Int J Mol Sci. 2024 May 20;25(10):5564. doi: 10.3390/ijms25105564 (PMC11122452; doi:10.3390/ijms25105564)
Supplement: Supplementary file 1 [file ijms-25-05564-s001.zip › Supplementary File.pdf]

Supplementary Materials:

Figure S1. Phylogenetic relationship of the *NCED* family in *Pisum sativum*, *Phaseolus vulgaris*, *Gossypium max*, *Arabidopsis thaliana*, *Gossypium hirsutum*, and *Oryza sativa* based on an unrooted evolutionary tree. The tree was constructed using the maximum likelihood method and the JTT+R5 model with 1,000 bootstrap replicates. The *NCED* genes are clustered into five subgroups (I–V) based on their evolutionary distances, as shown by different colors.

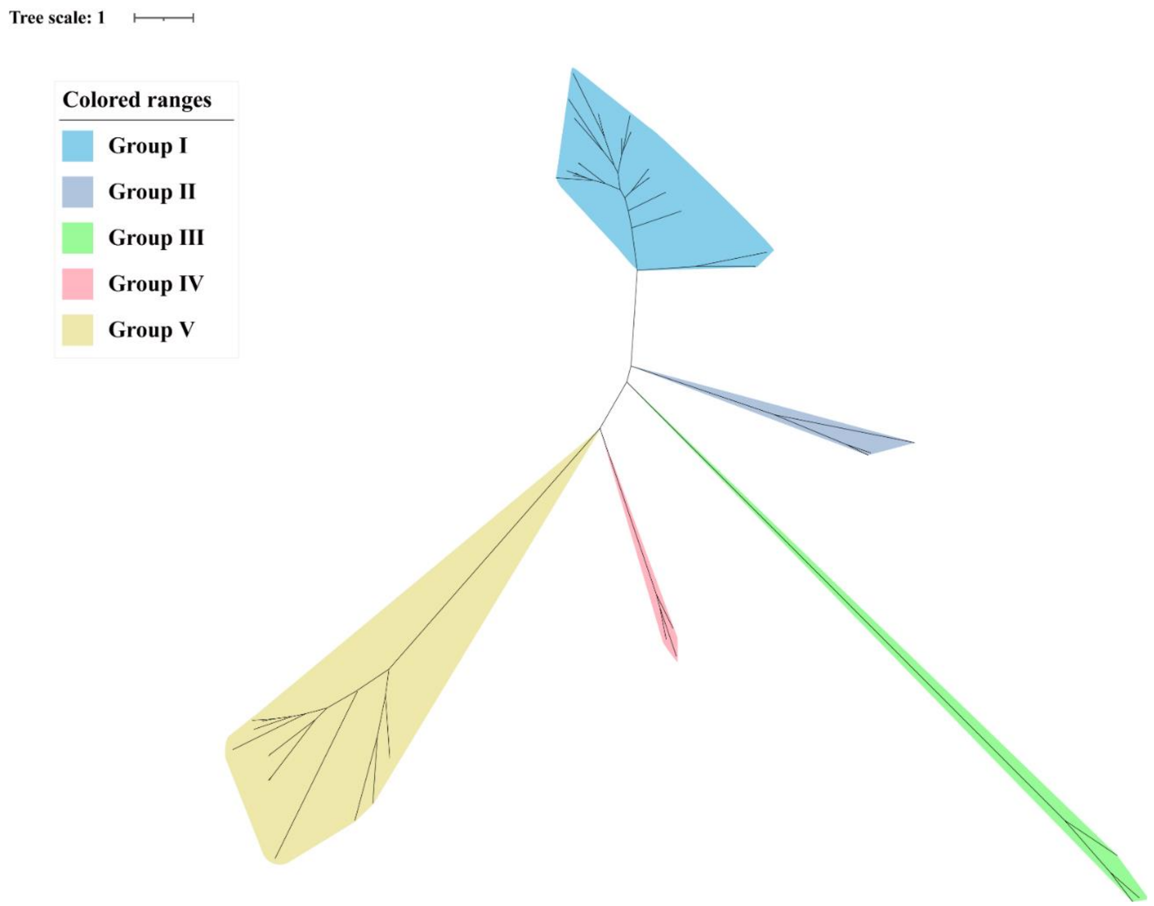

Figure S2. Different bar lengths in the chart represent the number of various functional cis-acting elements.

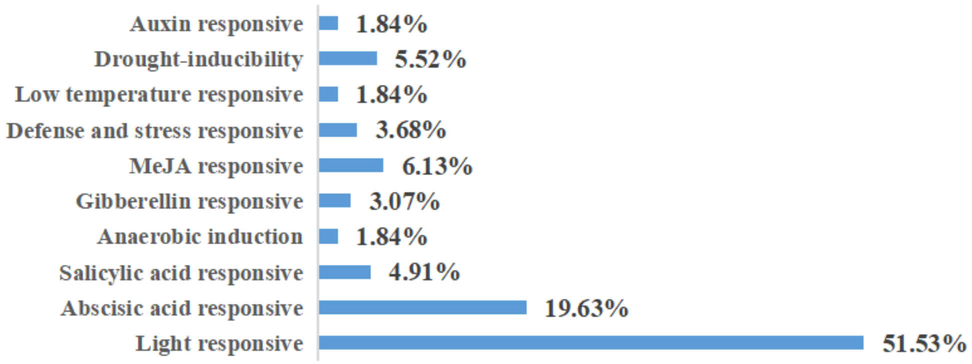

Figure S3. Three pairs of tandem duplicated genes (*AhNCED1* with *AhNCED2*, and *AhNCED3* with *AhNCED4*, as well as *AhNCED6* with *AhNCED7*).

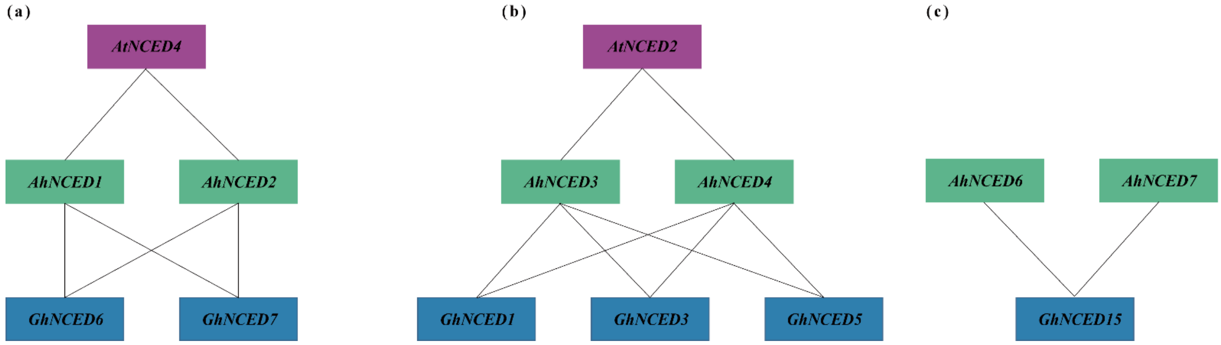

Figure S4. Protein structure simulation diagrams of the AhNCED3-8 proteins. Blue denotes alpha helices, while purple indicates beta helices. The pocket amino acids are represented in both sticks' and spheres' formats.

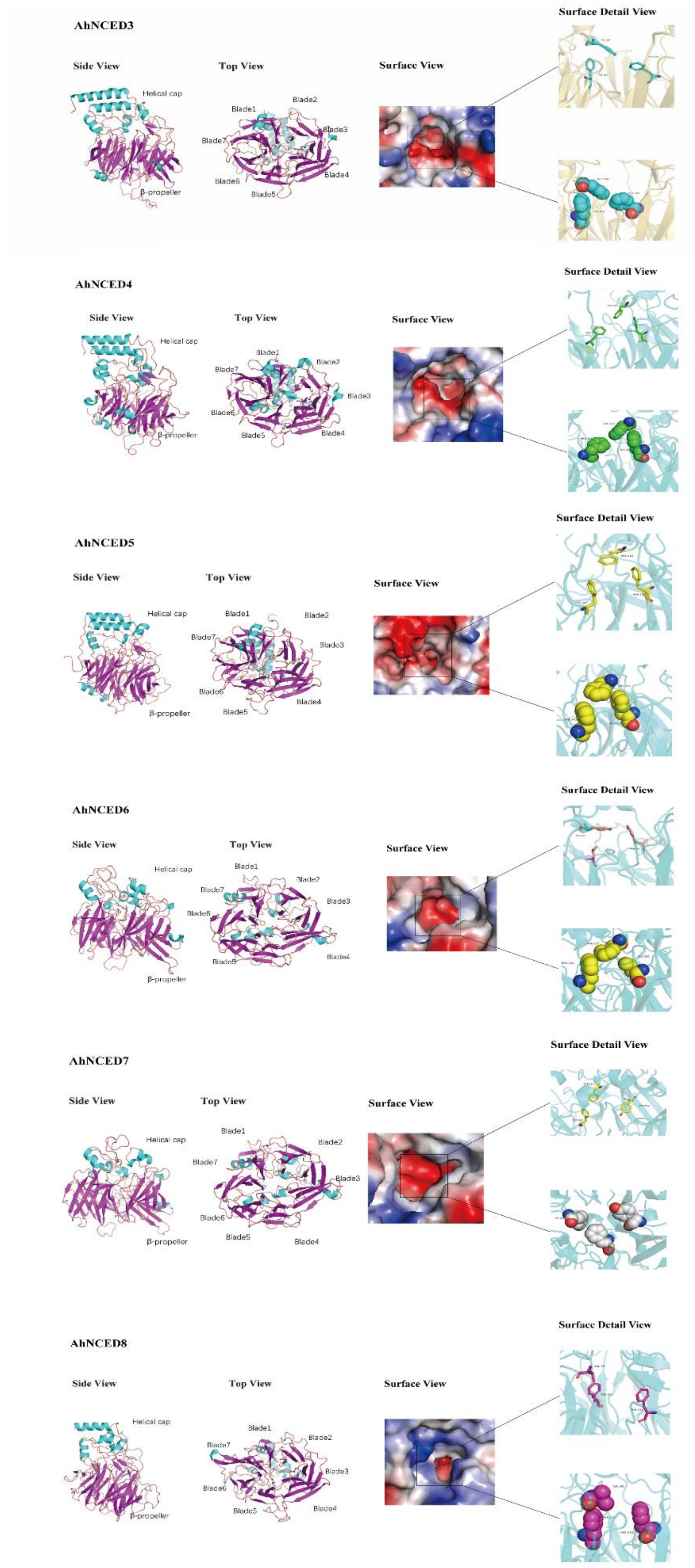

Table S1. Position information of cis-acting elements in the promoter of *NCED* gene in peanut.

Gene Name	Promoter Position	Promoter Name
AhNCED1	1637	salicylic acid responsive
AhNCED1	33	MeJA responsive
AhNCED1	652	light responsive
AhNCED1	654	light responsive
AhNCED1	714	light responsive
AhNCED1	1228	light responsive
AhNCED1	1712	light responsive
AhNCED1	524	gibberellin responsive
AhNCED1	730	anaerobic induction
AhNCED1	1172	anaerobic induction
AhNCED1	1823	anaerobic induction
AhNCED1	33	MeJA responsive
AhNCED1	652	abscisic acid responsive
AhNCED1	654	abscisic acid responsive
AhNCED1	655	abscisic acid responsive
AhNCED1	715	abscisic acid responsive
AhNCED1	1228	abscisic acid responsive
AhNCED1	1229	abscisic acid responsive
AhNCED1	1712	abscisic acid responsive
AhNCED1	1713	abscisic acid responsive
AhNCED1	654	light responsive
AhNCED1	1228	light responsive
AhNCED1	1712	light responsive
AhNCED1	83	light responsive
AhNCED1	1024	light responsive
AhNCED1	1304	light responsive
AhNCED1	1324	light responsive
AhNCED1	1334	light responsive
AhNCED1	1376	light responsive
AhNCED1	1386	light responsive
AhNCED1	1390	light responsive
AhNCED1	1420	light responsive
AhNCED1	1930	light responsive
AhNCED1	844	light responsive
AhNCED2	1079	light responsive
AhNCED2	1354	light responsive
AhNCED2	1358	light responsive
AhNCED2	1378	light responsive
AhNCED2	1388	light responsive
AhNCED2	1431	light responsive
AhNCED2	1449	light responsive
AhNCED2	727	light responsive
AhNCED2	1740	light responsive
AhNCED2	916	light responsive
AhNCED2	725	abscisic acid responsive
AhNCED2	727	abscisic acid responsive

AhNCED2	728	abscisic acid responsive
AhNCED2	788	abscisic acid responsive
AhNCED2	1740	abscisic acid responsive
AhNCED2	1741	abscisic acid responsive
AhNCED2	1666	salicylic acid responsive
AhNCED2	803	anaerobic induction
AhNCED2	1227	anaerobic induction
AhNCED2	1851	anaerobic induction
AhNCED2	597	gibberellin responsive
AhNCED2	725	light responsive
AhNCED2	727	light responsive
AhNCED2	787	light responsive
AhNCED2	1740	light responsive
AhNCED3	225	gibberellin responsive
AhNCED3	426	defense and stress responsive
AhNCED3	558	defense and stress responsive
AhNCED3	537	light responsive
AhNCED3	1742	light responsive
AhNCED3	79	light responsive
AhNCED3	1597	light responsive
AhNCED3	1296	light responsive
AhNCED3	56	abscisic acid responsive
AhNCED3	58	abscisic acid responsive
AhNCED3	538	abscisic acid responsive
AhNCED3	1742	abscisic acid responsive
AhNCED3	1743	abscisic acid responsive
AhNCED3	280	drought-inducibility
AhNCED3	1343	drought-inducibility
AhNCED3	1576	drought-inducibility
AhNCED3	1981	drought-inducibility
AhNCED3	1530	salicylic acid responsive
AhNCED3	58	light responsive
AhNCED3	1742	light responsive
AhNCED3	547	anaerobic induction
AhNCED3	1352	anaerobic induction
AhNCED3	1109	light responsive
AhNCED3	1120	light responsive
AhNCED3	1235	light responsive
AhNCED3	996	low temperature responsive
AhNCED3	1424	low temperature responsive
AhNCED3	120	MeJA responsive
AhNCED3	120	MeJA responsive
AhNCED4	464	light responsive
AhNCED4	988	light responsive
AhNCED4	1667	light responsive
AhNCED4	353	defense and stress responsive

AhNCED4	485	defense and stress responsive
AhNCED4	1009	light responsive
AhNCED4	1035	light responsive
AhNCED4	1043	light responsive
AhNCED4	1170	light responsive
AhNCED4	152	gibberellin responsive
AhNCED4	1359	low temperature responsive
AhNCED4	1231	light responsive
AhNCED4	1530	light responsive
AhNCED4	50	MeJA responsive
AhNCED4	207	drought-inducibility
AhNCED4	1278	drought-inducibility
AhNCED4	1509	drought-inducibility
AhNCED4	1981	drought-inducibility
AhNCED4	465	abscisic acid responsive
AhNCED4	988	abscisic acid responsive
AhNCED4	1667	abscisic acid responsive
AhNCED4	1668	abscisic acid responsive
AhNCED4	1463	salicylic acid responsive
AhNCED4	474	anaerobic induction
AhNCED4	1287	anaerobic induction
AhNCED4	50	MeJA responsive
AhNCED4	1667	light responsive
AhNCED5	1865	light responsive
AhNCED5	1867	light responsive
AhNCED5	1941	light responsive
AhNCED5	1967	light responsive
AhNCED5	1968	light responsive
AhNCED5	116	MeJA responsive
AhNCED5	496	salicylic acid responsive
AhNCED5	1004	light responsive
AhNCED5	1870	abscisic acid responsive
AhNCED5	1968	abscisic acid responsive
AhNCED5	116	MeJA responsive
AhNCED5	511	light responsive
AhNCED5	1244	light responsive
AhNCED5	1474	light responsive
AhNCED5	1556	light responsive
AhNCED5	1630	light responsive
AhNCED5	1869	light responsive
AhNCED6	1592	defense and stress responsive
AhNCED6	1143	light responsive
AhNCED6	1246	auxin responsive
AhNCED6	1316	light responsive
AhNCED6	1346	light responsive
AhNCED6	1545	light responsive
AhNCED6	1666	light responsive

AhNCED6	1094	gibberellin responsive
AhNCED6	862	abscisic acid responsive
AhNCED6	864	abscisic acid responsive
AhNCED6	1143	abscisic acid responsive
AhNCED6	1397	abscisic acid responsive
AhNCED6	960	salicylic acid responsive
AhNCED6	430	anaerobic induction
AhNCED6	864	light responsive
AhNCED6	1397	light responsive
AhNCED7	720	light responsive
AhNCED7	1249	light responsive
AhNCED7	1279	light responsive
AhNCED7	1632	light responsive
AhNCED7	1642	light responsive
AhNCED7	1178	auxin responsive
AhNCED7	1996	defense and stress responsive
AhNCED7	804	light responsive
AhNCED7	1075	light responsive
AhNCED7	805	abscisic acid responsive
AhNCED7	1075	abscisic acid responsive
AhNCED7	1330	abscisic acid responsive
AhNCED7	1330	light responsive
AhNCED7	423	salicylic acid responsive
AhNCED7	605	salicylic acid responsive
AhNCED8	1740	MeJA responsive
AhNCED8	484	anaerobic induction
AhNCED8	1713	anaerobic induction
AhNCED8	1777	anaerobic induction
AhNCED8	76	light responsive
AhNCED8	101	light responsive
AhNCED8	1965	light responsive
AhNCED8	1972	light responsive
AhNCED8	1246	auxin responsive
AhNCED8	1740	MeJA responsive
AhNCED8	943	drought-inducibility

Table S2. Fold change of *AhNCED* genes expression in drought-tolerant variety (NH5) under different time points of drought stress treatment (0h, 4h, 8h, and 24h), represented in FPKM (Fragments Per Kilobase Million). Only the *NCED* genes with FPKM values above 10 in at least one sample are expressed in the fold change values of this heatmap to avoid over-emphasis changes in genes with low expression levels.

	4h	8h	24h
AhNCED1	17.67	9.26	7.49
AhNCED2	18.40	10.15	9.83

Table S3. Fold change of *AhNCED* genes expression in drought-sensitive variety (FH18) under different time points of drought stress treatment (0h, 4h, 8h, and 24h), represented in FPKM (Fragments Per Kilobase Million). Only the *NCED* genes with FPKM values above 10 in at least one sample are expressed in the fold change values of this heatmap to avoid over-emphasis changes in genes with low expression levels.

	4h	8h	24h
AhNCED1	25.53	12.21	6.69
AhNCED2	37.67	18.56	10.95

Table S4. Primers for quantitative PCR.

Gene	Forward Primer	Reverse Primer
AhNCED1	TCACCCCCTGCTACTTACTTC	TGAGGATGGTGTTTGAGGTGA
AhNCED2	TCACCCCCTGCTACTTACTTC	TGAGGATGGTGTTTGAGGTGA
AhNCED3	GTTGACCCAAGCACACACCA	AGAAAGGGCGTGACAGTTGA
AhNCED4	GTTGACCCAAGCACACACCA	AGAAAGGGCGTGACAGTTGA
AhNCED5	CGAGTTACGACCCACATGCA	GATGCCAACCCTTTGCTTGG
AhNCED6	ACCTCACTCTCCTACACTCCC	TAGAAACAACCATGCTTCTTGGA
AhNCED7	ACCTCACTCTCCTACACTCCC	TAGAAACAACCATGCTTCTTGGA
AhNCED8	TCCCCACATATATACACTTCTTCAT	AGTTGCCACAATTTTCGGGA
AhActin	TTGGAATGGGTCAGAAGGATGC	AGTGGTGCCTCAGTAAGAAGC
