# Supplementary figures and images for: Identification and Expression Profile of NCED Genes in Arachis hypogaea L. during Drought Stress
Source: Int J Mol Sci. 2024 May 20;25(10):5564. doi: 10.3390/ijms25105564 (PMC11122452; doi:10.3390/ijms25105564)

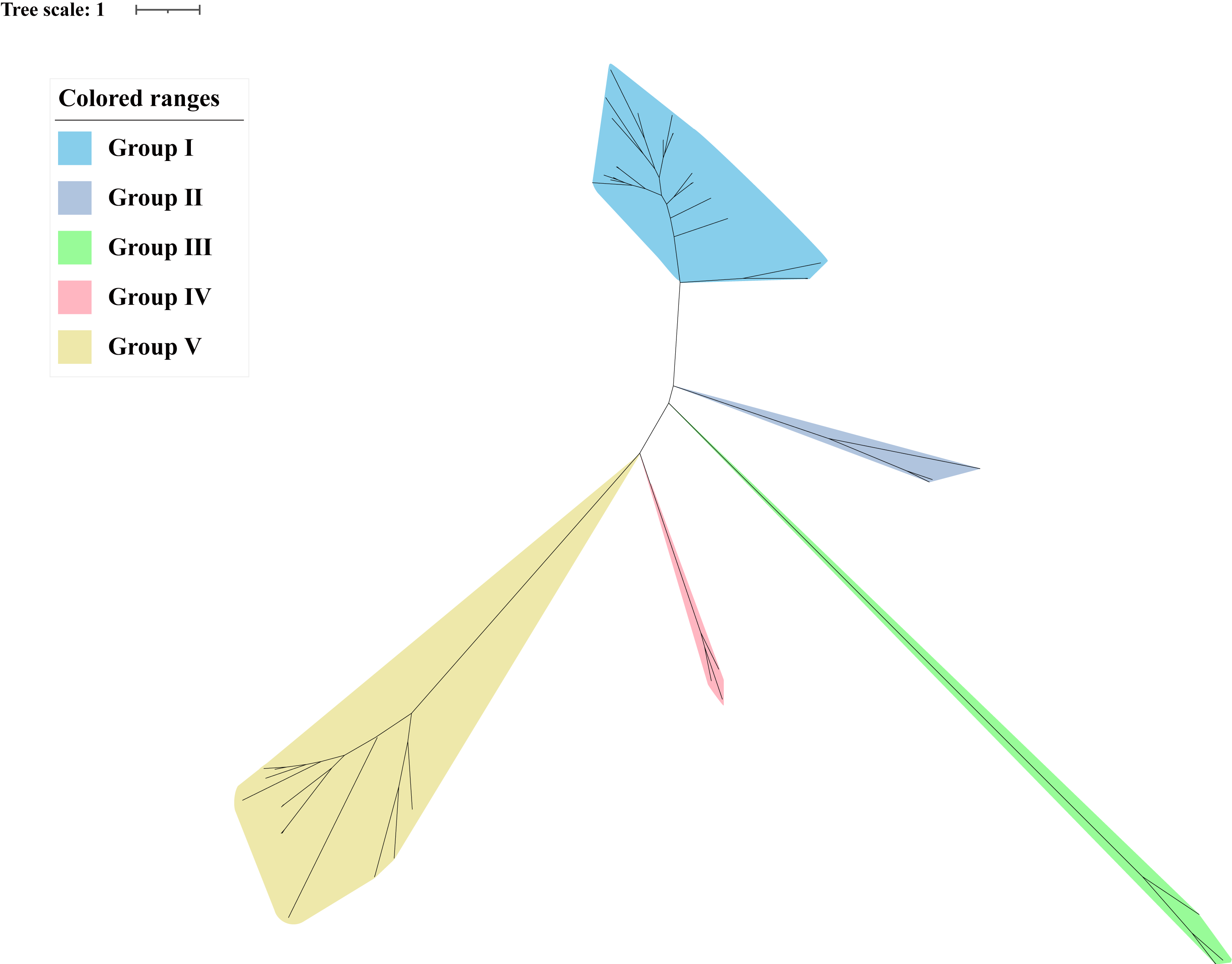

Supplement: Supplementary file 1 [file ijms-25-05564-s001.zip › Figure S1.tif]

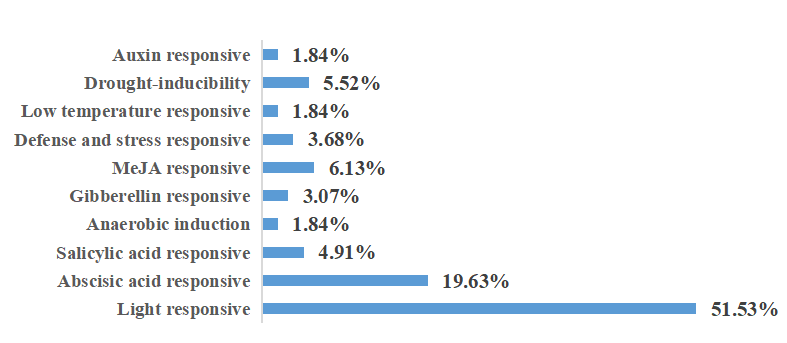

Supplement: Supplementary file 1 [file ijms-25-05564-s001.zip › Figure S2.tif]

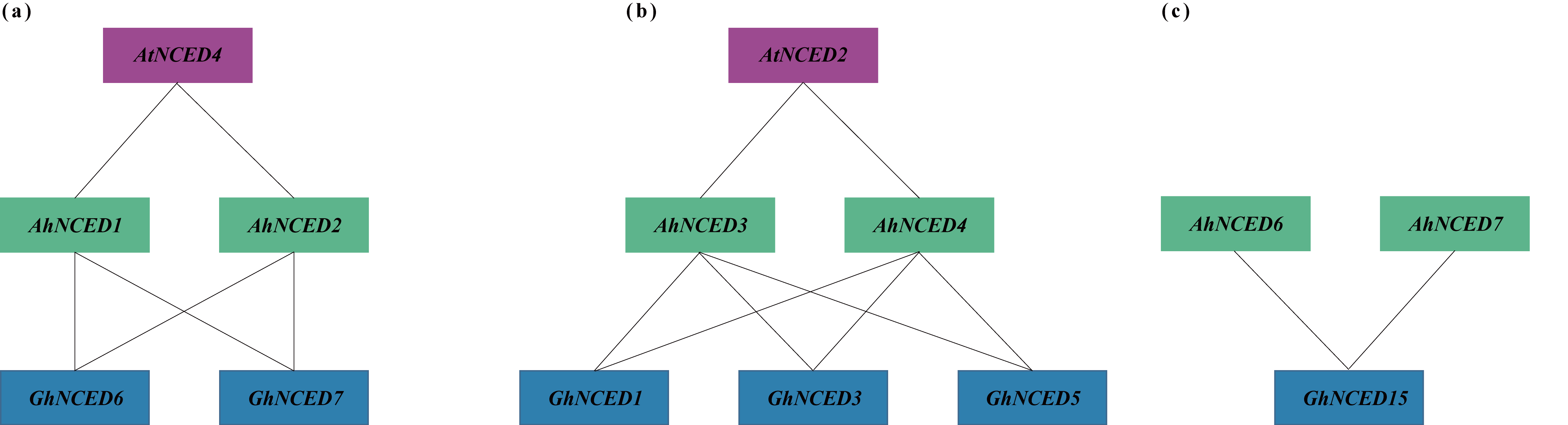

Supplement: Supplementary file 1 [file ijms-25-05564-s001.zip › Figure S3.tif]

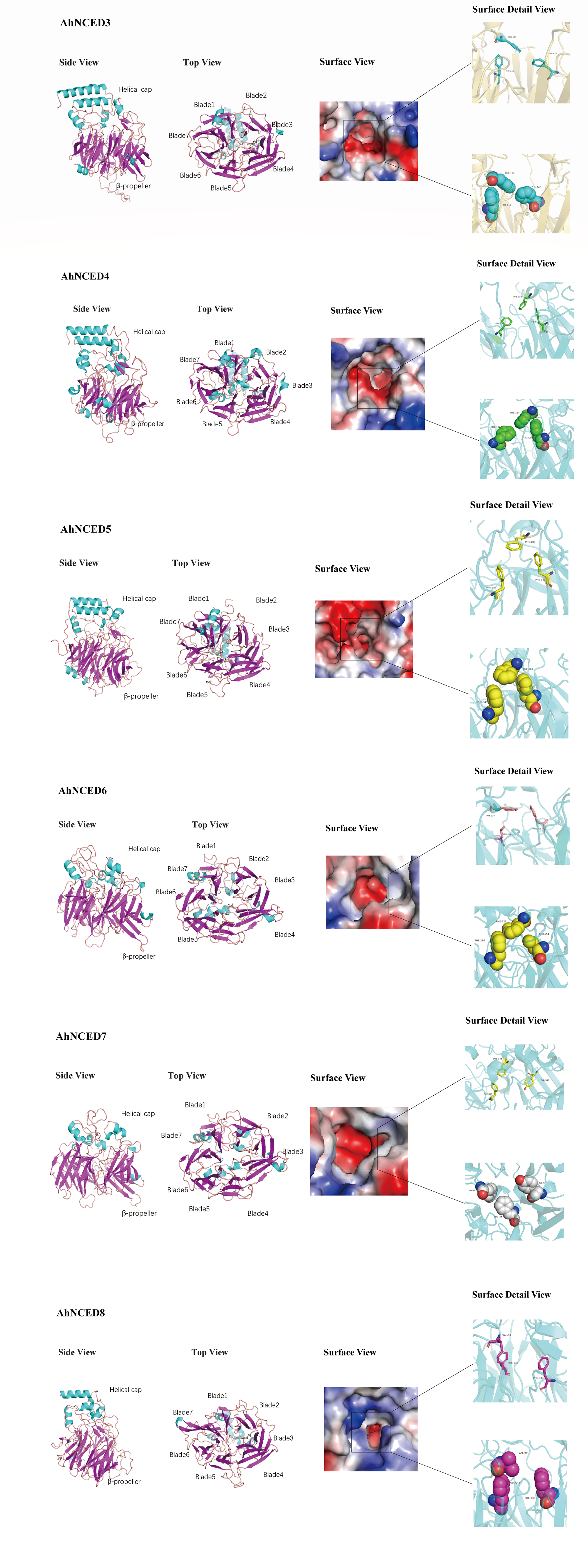

Supplement: Supplementary file 1 [file ijms-25-05564-s001.zip › Figure S4.tif]

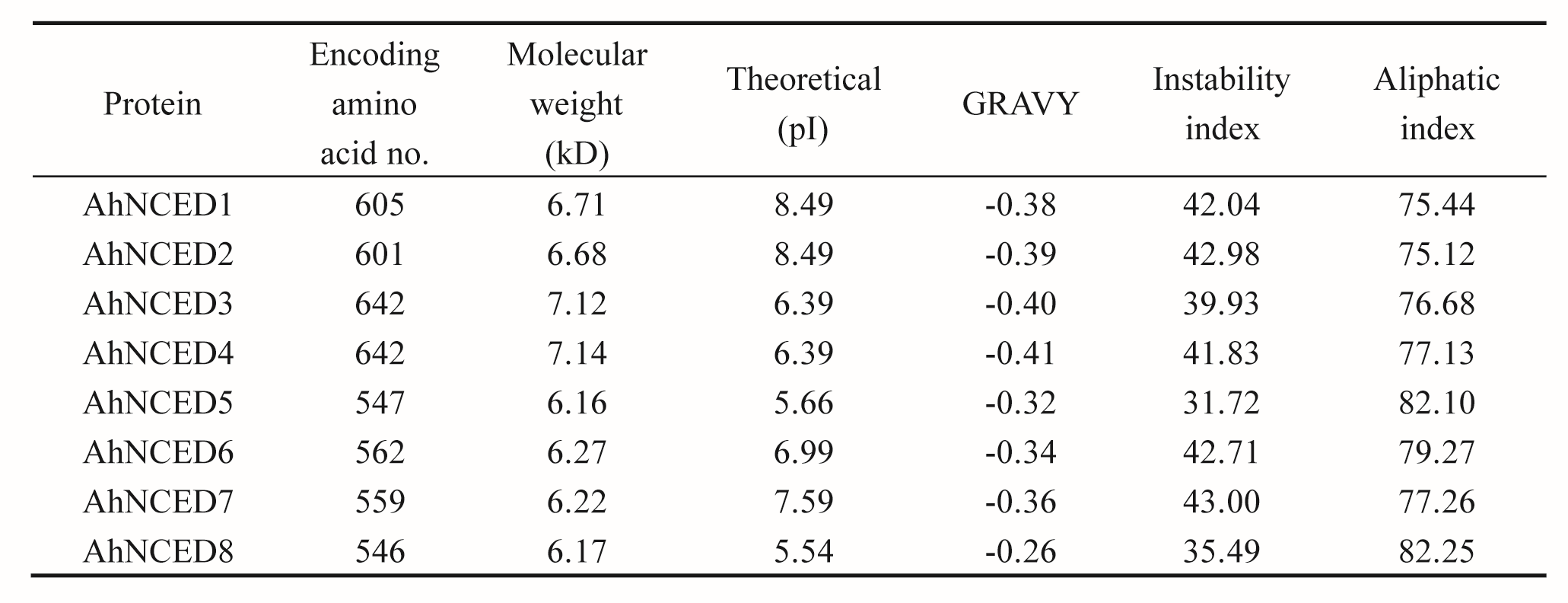

Supplement: Supplementary file 1 [file ijms-25-05564-s001.zip › Table 1.tif]
